# Chromosomal Evolution of the Talpinae

**DOI:** 10.3390/genes14071472

**Published:** 2023-07-19

**Authors:** Larisa S. Biltueva, Nadezhda V. Vorobieva, Natalya A. Lemskya, Polina L. Perelman, Vladimir A. Trifonov, Victor V. Panov, Alexey V. Abramov, Shin-ichiro Kawada, Natalya A. Serdukova, Alexandr S. Graphodatsky

**Affiliations:** 1 Institute of Molecular and Cellular Biology SB RAS, Lavrentiev Ave., 8/2, 630090 Novosibirsk, Russia; vorn@mcb.nsc.ru (N.V.V.); lemnat@mcb.nsc.ru (N.A.L.); perelmanp@mcb.nsc.ru (P.L.P.); vlad@mcb.nsc.ru (V.A.T.); serd@mcb.nsc.ru (N.A.S.); graf@mcb.nsc.ru (A.S.G.); 2 Institute of Systematics and Ecology of Animals SB RAS, Frunze st.11, 630091 Novosibirsk, Russia; panovv53@mail.ru; 3 Zoological Institute RAS, 199034 Saint Petersburg, Russia; a.abramov@mail.ru; 4 Joint Vietnamese-Russian Tropical Research and Technological Centre, Nguyen Van Huyen, Nghia Do, Cau Giay, Hanoi 650000, Vietnam; 5 Department of Zoology, National Museum of Nature and Science, 4-1-1, Amakubo, Tsukuba 305-0005, Ibaraki, Japan; kawada@kahaku.go.jp

**Keywords:** *Talpinae* species, chromosome evolution, comparative chromosome painting

## Abstract

In recent years, the number of mole species with species status confirmed by genetic methods has been continuously increasing. Unfortunately, cytogenetic data are not yet available for all species. Here, for the first time, a GTG-banded karyotype of the small-toothed mole from Vietnam, *Euroscaptor parvidens*, a representative of the Eastern clade of the genus *Euroscaptor*, has been described. Through comparative analysis of available *Euroscaptor (Euroscaptor parvidens*, *Euroscaptor klossi*, and *Euroscaptor malayana)* and *Oreoscaptor (Oreoscaptor mizura)* karyotypes, we found cytogenetic signatures for each of the studied species. Zoo-FISH with sorted chromosomes of the Siberian mole (*Talpa altaica)* on chromosome sets of the small-toothed mole (*E. parvidens)*, the small Japanese mole (*Mogera imaizumii*) from the closely related genus, and the Japanese shrew mole (*Urotrichus talpoides)* from the tribe *Urotrichini* made it possible to identify syntenic regions between these species. We propose a possible ancestral karyotype of the tribe and, based on it, traced the features of chromosomal rearrangements accompanying the divergence of moles. The low rates of chromosomal evolution within the species of the genus *Talpa—T. altaica* and *T. europaea*—and the high rates of karyotypic reshuffling within the Asian genera of the tribe were confirmed. The karyotype of the Japanese mountain mole *O. mizura* seems to be the most conserved among the Asian moles. The most frequently occurring types of chromosomal rearrangements in moles are the pericentric inversions and amplification of heterochromatin. The pericentric inversions on four pairs of autosomes are shared between the closely related genera *Euroscaptor*, *Oreoscaptor*, and *Mogera*, while many more apomorphic rearrangements have occurred in each lineage additionally. The highest rate of chromosomal changes, with five rearrangements occurring over approximately 7 million years, was recorded in the lineage of the small-toothed mole.

## 1. Introduction

The subterranean species represent an interesting model to study the patterns and rates of evolution due to their restricted dispersal and concerted evolution of drastic adaptations to the underground habitat. For a long time, there has been a question regarding whether chromosomal changes occur differently in subterranean species compared to surface-dwelling mammalian species [1]. Here, we examine the evolutionary dynamics of chromosomal complements within the subfamily of Eurasian moles that mainly live in underground environments.

The family of moles (Talpidae) is a diverse group of underground insectivores in the order Eulypotyphla. The subfamily Talpinae includes Old World moles, desmans, and shrew moles predominantly found in Eurasia. Talpinae is further classified into five tribes. Notably, two sister tribes within the subfamily Talpinae are a specious and widely distributed tribe Talpini (6 genera, 22 species) and tribe Urotrichini (two monospecific genera *Urotrichus* and *Dymecodon*) (Table S1). The underground lifestyle of the Talpini results in similar physical traits of moles and makes it difficult to differentiate them, leading to the underestimation of the species diversity [2]. Among Talpini, the genus *Talpa* is widely distributed from Western Europe to Asia. The other five genera diverged soon after their separation from the common ancestor with *Talpa* and have limited ranges in Eastern Asia: *Mogera* (5–9 species), *Euroscaptor* (6–8 species), monospecific *Scaptochirus*, *Parascaptor*, and *Oreoscaptor* [3,4,5].

The phylogeny of the Talpini tribe is now well established. Genetic research provided good support for phylogenies based on morphological data [2,3,6]. High genetic differentiation within the most successful genus *Talpa* allowed it to identify four clades with a strict geographical reference for the European and Caucasian group of species, as well as the “davidiana” group, whereas the re-colonization of Europe from Asia was shown [7]. Nuclear sequence analysis demonstrated the nested position of *Talpa. altaica* within *Talpa* as a separate Siberian clade. However, previous morphological and mitochondrial data indicated its more isolated position [8] and even an elevation to a separate genus rank, *Asioscalops altaica* [9]. The genus *Euroscaptor* contains two strongly divergent clades: the Western one with the type species *Euroscaptor klossi*, including *Euroscaptor malayana* and *Euroscaptor longirostris sensu lato*, and the Eastern one with *Euroscaptor parvidens sensu lato* and the recently discovered species *Euroscaptor subanura* [4,10,11,12]. The taxonomy of *E. parvidens* and *E. longirostris* has not been definitively resolved [3]; therefore, it was proposed to distinguish two additional new species within *E. longirostris sensu lato*: *Euroscaptor orlovi* and *Euroscaptor kuznetsovi*, and a new subspecies of *Euroscaptor parvidens ngoclinhensis* within *E. parvidens* [4]. Recently, Bui et al. [13] suggested that moles from the Dalat Plateau (southern Vietnam) and those from the Kontum Plateau (central Vietnam) may represent different species, *Euroscaptor parvidens* and *E. ngoclinhensis*, respectively.

The only island species of *Euroscaptor sensu lato*, *Euroscaptor mizura*, claims the status of a separate new genus *Oreoscaptor*, being closer in its characteristics to the sister genus *Mogera* [4,10,11,14]. The genus *Mogera* includes nine species, with a widespread continental distribution (*Mogera robusta* and *Mogera latouchei*) and island species with a limited range (*Mogera wogura*, *Mogera imaizumii*, *Mogera tokudae*, *Mogera etigo*, *Mogera uchidai*, *Mogera insularis*, and *Mogera kanoana*) and its monophyly is indisputable [3,5,15]. The taxonomic status of many *Mogera* species has been established recently based on different characters, including karyotypic data. For example, *M. tokudae* and *M. etigo* previously represented two chromosomal races of the same *M. tokudae* specie, differing by three pericentric inversions [16]; at present, however, they are considered as two separate species. At the same time, genetic and morphological data were sufficient to separate *M. insularis* and *M. kanoana*, which have similar karyotypes [17]. The taxonomic status of the continental and insular species *M. robusta* and *M. wogura* was resolved recently [5].

Karyotypic studies have shown that chromosome sets within the genus *Talpa* are sufficiently conserved: the differences are mainly due to a variation in the size of heterochromatin blocks on several pairs of autosomes [18,19,20,21]. The Asian genera of the tribe Talpini show higher rates of chromosomal changes affecting the euchromatic part of the genome. For example, within the genus *Mogera*, four inversions separate the Korean (*M. robusta)* and Japanese populations of *M. wogura* [16]. At the same time, different species of *Mogera* may have similar karyotypes, such as *M. etigo* and *M. imaizumii*, and the identity between the karyotypes of *M. wogura* and *O. mizura* [16] indicates that they have retained the same chromosome set from their common ancestor. Similar karyotypes of the Taiwanese species *M. kanoana* and *M. insularis* are characterized by a smaller number of chromosomes (2n = 32), while in other species of the genus 2n = 36 [17]. The chromosomal evolution of the genus *Euroscaptor* is traced in detail by studies of G-banded karyotypes of *E. klossi* and *E. malayana* and the Japanese mountain mole (*Oreoscaptor mizura)* (formerly treated as *Euroscaptor* species). Comparative analysis revealed a reciprocal translocation that separates the karyotypes of *O. mizura* from the Malaysian mole *E. malayana* [22]. A reciprocal translocation followed by a pericentric inversion separates the karyotypes of the Japanese mountain mole and Kloss’ mole [23].

In the tribe of the Japanese shrew moles, Urotrichini (which is closely related to Talpini), there are two monospecific genera: *Dymecodon* and *Urotrichus*, the karyotypic relationships between which have been studied in detail. There are the Eastern and Western forms of *Urotrichus talpoides* that differ by the pericentric inversion of chromosome pair 14, accompanied by the accumulation of heterochromatin in the Eastern form [24]. According to craniological data, *Dymecodon pilirostris* is considered more primitive, and its karyotype is similar to the karyotype of the Western form of *U. talpoides* [25]. 

Molecular cytogenetic analysis in the Talpini tribe was carried out only for two species of the genus *Talpa*: *Talpa europaea* (TEUR) and *Talpa altaica* (TALT) [26,27]. Using human flow sorting-derived painting probes, it was shown that the euchromatin segments of all chromosomes of both species are similar and have the same distribution of segments along the chromosomes, with the exception of the homeologous chromosome TEUR13, which underwent a pericentric inversion and/or centromeric shift. Additional heterochromatic arms changed the morphology of homeologous chromosomes TALT 1/TEUR 9 and TALT 6/TEUR 1. Such a high level of chromosome conservation is surprising for an early diverged species characterized by a high genetic diversity [7]. Zoo-FISH analysis of data from seven species representing all three insectivoran families—moles, hedgehogs, and shrews—revealed conserved blocks and syntenic associations of chromosomes preserved from the putative eutherian common ancestor. Comparing the syntenic associations between insectivoran families allowed us to assess the degree of their evolutionary variability, and it demonstrated that moles’ karyotypes are the most conserved among insectivores [27].

In this paper, we describe for the first time a GTG-banded karyotype of the small-toothed mole (*E. parvidens)*. We traced chromosomal transformations within the genus *Euroscaptor* by comparing the karyotype of *E. parvidens* with other karyotypes within the genus. We assess the level of karyotypic transformations within the entire Talpinae subfamily by Zoo-FISH of chromosome-specific painting probes of the Siberian mole (*T. altaica)* onto metaphase chromosomes of three representatives of the Talpinae subfamily: *E. parvidens* and *M. imaizumii* (the Talpini tribe); and Urotrichus talpoides (the Urotrichini tribe).

## 2. Materials and Methods

Metaphase chromosome spreads were prepared from the primary fibroblast culture of a male individual of *Euroscaptor parvidens* (EPAR). The specimen comes from southern Vietnam, Phu Yen Province. Cell culture and metaphase preparations were obtained following the conventional methods as described previously [28]. GTG-banding and CBG-banding followed Seabright [29] and Sumner [30], respectively. Chromosomes in the karyotype of *E. parvidens* were arranged by size and according to centromere positions. Karyotypes of *Urotrichus talpoides* (UTAL) and *Mogera imaizumii* (MIMA) were arranged according to Kawada et al. [16,25]. *Talpa altaica* chromosome-specific painting probes were made by P.C.M. O’Brien and V. Trifonov from flow-sorted chromosomes [31,32]. FISH was performed following GTG-banding of metaphase chromosomes according to published protocols [28,33]. The karyotypes of the individuals examined here have been described previously: *T. altaica* [27]; *T. europaea* [26]; *M. imaizumii* [16]; *U. talpoides* [25,34]; *O. mizura* [16]; *E. klossi* [23]; *E. malayana* [35]. 

## 3. Results

### 3.1. Description of the Karyotype of the Small-Toothed Mole (Euroscaptor parvidens) from Vietnam

**Figure 1:**
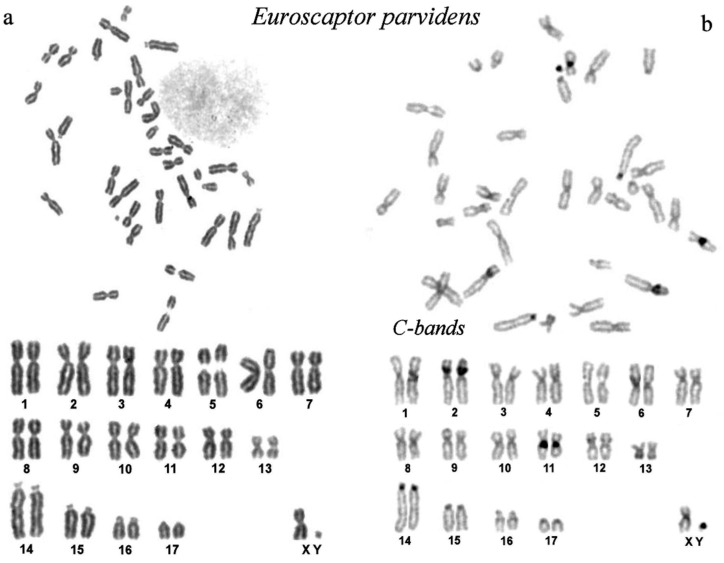
The karyotype of small-toothed mole from Vietnam, *Euroscaptor parvidens*, 2n = 36. (**a**) Routine Giemsa chromosome staining: top—complete metaphase plate, bottom—the karyotype. Note the secondary constriction on chromosome 5, likely site of nuclear organizing region (NOR). (**b**) Constitutive heterochromatin by CBG staining: top—complete metaphase plate, bottom—the karyotype. Note prominent heterochromatin bands on chromosomes 2, 11, 14, and Y.

The karyotype of the small-toothed mole (2n = 36) consists of 17 pairs of autosomes: 13 pairs of metacentrics, 3 pairs of subtelocentrics, and 1 pair of acrocentrics; the submetacentric X chromosome and the smallest chromosome in the complement is the Y chromosome (Figure 1a). 

Large heterochromatin blocks are located in the pericentromeric regions of the short arm of chromosome 2 and the long arm of chromosome 11. Small blocks of C-heterochromatin are located in the pericentromeric regions of the large acrocentric chromosome 14 and the tiny Y chromosome (Figure 1b). 

**Figure 2:**
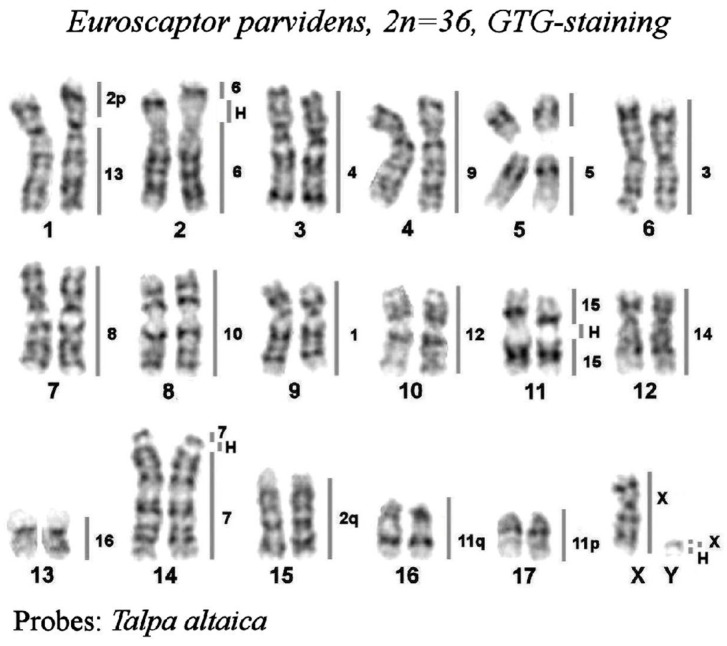
The karyotype of small-toothed mole (Euroscaptor parvidens) from Vietnam 2n = 36, GTG-staining. The lines and numbers on the right delineate homologous chromosome segments revealed by mapping the set of *Talpa altaica* chromosome painting probes by FISH. “H” designates the heterochromatic regions.

The GTG-banded karyotype of the small-toothed mole is shown in Figure 2. The detailed description of homologous chromosome segments of Talpa altaica, revealed by FISH will be given in Section 3.3.2. 

### 3.2. Comparison of G-Banded Chromosomes of Euroscaptor Species

**Figure 3:**
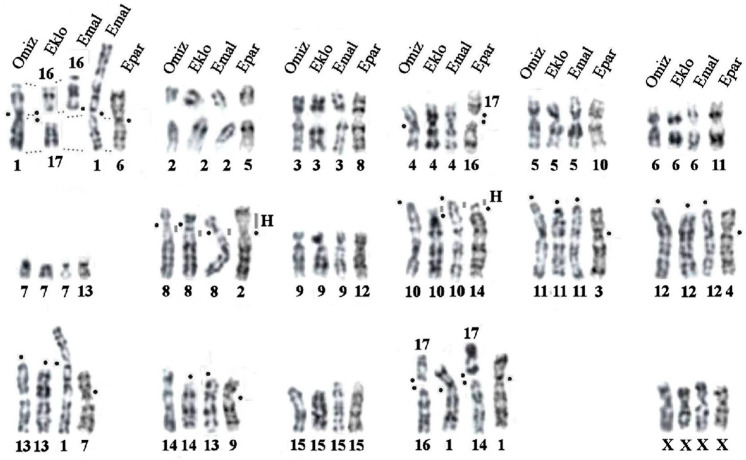
Comparative analysis tracing chromosome homologies between G-banded karyotypes of four Asian mole species from *Euroscaptor* and *Oreoscaptor* genera: *E. parvidens* (Epar), *E. klossi* (Eklo), *E. malayana* (Emal), and the Japanese mountain mole, *Oreoscaptor mizura* (Omiz) [16,23,35]. “H”—designates constitutive heterochromatin blocks. Black dots designate centromere positions.

A comparative chromosomal analysis of *E. parvidens* with available G-banded karyotypes of *Euroscaptor* species (*E. klossi, E. malayana*) and the Japanese mountain mole, *Oreoscaptor mizura* [16,23,35], was performed (Figure 3).

The chromosomes of these species were arranged relative to the Japanese mountain mole, *O. mizura,* nomenclature (Table 1). Chromosomal analysis showed the following:

- Seven autosomal pairs of the small-toothed mole (chromosomes 5, 8, 10–13, and 15) are homologous to chromosomes 2–7, 10, and 15, respectively, in the all compared species, as well as their X chromosomes;
- Accumulation of repeats in the short arm changed the morphology of the submetacentric chromosome 2 of *E. parvidens* compared to the homologous chromosome 8 of other species;
- Four metacentric chromosomes of the small-toothed mole (chromosomes 3, 4, 7, and 9) underwent pericentric inversions in contrast to the homologous acrocentric 11, 12, 13, and 14, respectively, of other species;
- A small inversion of the pericentromeric heterochromatin distinguishes the subtelocentric chromosomes 14 of *E. parvidens*, 10 of *E. klossi* (EKLO), and the acrocentric chromosome 10 of both *O. mizura* (OMIZ) and *E. malayana* (EMAL), respectively;
- Centric fusions/fissions separate chromosomes/chromosomal arms:
  (a) OMIZ 1 and EPAR 6 with EKLO 16+17 and EMAL 1p+16;
  (b) OMIZ 4, EKLO 4, and EMAL 4 with EPAR 16+17;
  (c) OMIZ 17+16 and EMIC 17+14 with EKLO 1 and EPAR 1.

The results of the analysis are summarized in Table 1.

### 3.3. Chromosome Painting

#### 3.3.1. Assignment of the Peaks of the Flow-Sorted Karyotype

The 17 chromosomes of a female *T. altaica* were resolved into 14 peaks (Figures S1 and S2, Table S3). Eleven peaks each contained a single chromosome (TALT 1, 2, 3, 5 8, 10–12, 14–16), and three peaks included two chromosomes each (TALT 4+7, 6+9, 13+X). Therefore, the whole set of painting probes contains all 16 pairs of autosomes and the X chromosome. 

#### 3.3.2. Cross-Species Chromosome Painting

The whole chromosome set of the Siberian mole (*T. altaica*, TALT) painting probes was hybridized onto chromosomes of three moles from Southeast Asia: the small-toothed mole (*E. parvidens*, EPAR) and small Japanese mole (*M. imaizumii*, MIMI) from tribe Talpini and the Japanese shrew mole (*U. talpoides*, UTAL) from tribe Urotrichini. 

The chromosome painting delimited homologous chromosomal segments between species from different tribes and provided correspondence between the conserved chromosomal segments within the subfamily Talpinae. Additional rearrangements within the found segments, including pericentric inversions and centromeric shifts, were revealed using a comparative analysis of GTG-banded chromosomes.

##### Painting of the *E. parvidens* Karyotype with *T. altaica* Probes

**Figure 4:**
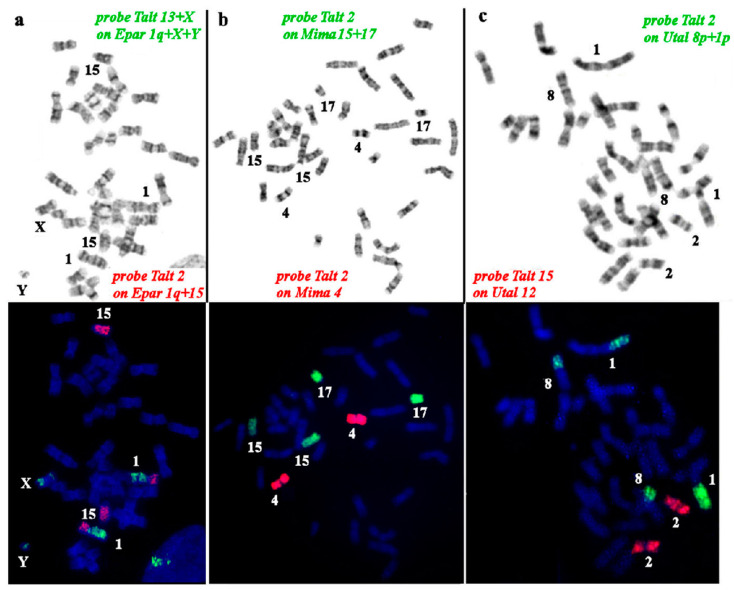
Comparative chromosome painting using a *Talpa altaica* (Talt) set of painting probes among several talpin mole species: (**a**). *Euroscaptor parvidens* (Epar), (**b**). *Mogera imaizumii* (Mima), (**c**). *Urotrichus talpoides* (Utal).

The hybridization results are presented as a map, where all *T. altaica* probes are assigned to the G-banded karyotype of *E. parvidens* (Figure 2). The Siberian mole painting probes delineated 20 homologous segments in the small-toothed mole karyotype. Fourteen Siberian mole chromosomes (TALT 1, 3–10, 12, 14–16, and X) were conserved in the karyotype of the small-toothed mole in toto. The metacentric chromosome TALT 11 underwent centromeric fission, and its arms are represented as acrocentrics EPAR 16 and EPAR 17. The large metacentric TALT 2 also splits into two segments, and the smallest segment then fused with TALT 13, forming the largest chromosome EPAR 1. The largest part of TALT 2 formed the acrocentric EPAR 15 (Figure 4a). The weak signal on EPAR 5p can be explained by the underrepresentation of the homologous chromosome of TALT5 in the painting probe. The presence of additional signals produced by both TALT1 and TALT6 on the EPAR 2p is explained by the presence in these areas of heterochromatin blocks of similar composition.

1. *Chromosome* painting of *Mogera imaizumii* with *T. altaica* probes

**Figure 5:**
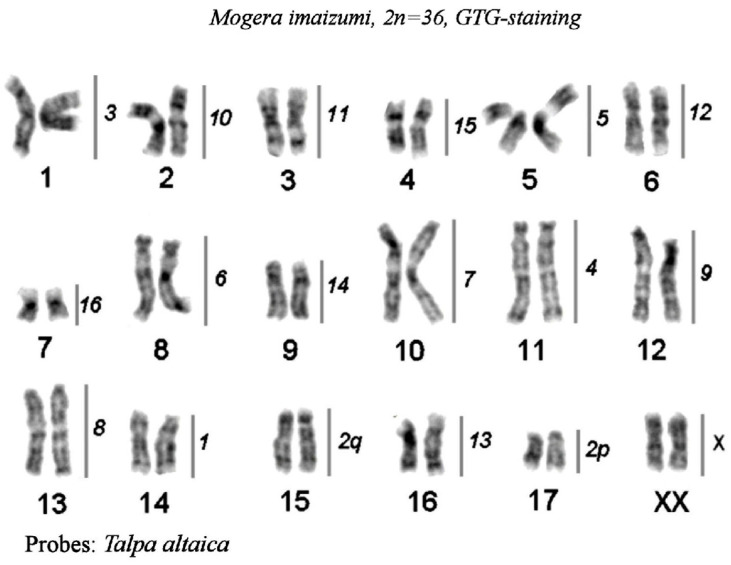
Comparative chromosome map between *M. imaizumii* and *T. altaica*. The karyotype of the small Japanese mole, *Mogera imaizumi*, 2n = 36, GTG-staining. The lines and numbers on the right delineate homologous chromosome segments revealed by FISH mapping of the set of *Talpa altaica* chromosome painting probes. *T. altaica* chromosome 2 is split in *M. imaizumii*.

The hybridization results are presented as a map on the G-banded karyotype of the small Japanese mole (*Mogera imaizumii*) (Figure 5). The Siberian mole painting probes delineated 18 homologous segments in the small Japanese mole karyotype. Almost all Siberian mole chromosomes (TALT 1, 3–16, and X) were entirely conserved in the karyotype of *M. imaizumii*; only one metacentric TALT 2 underwent fission into two segments which produced acrocentrics MIMI 15 and MIMI 17 (Figure 4b).

2. *Chromosome* painting between *Urotrichus talpoides* and *T. altaica*

**Figure 6:**
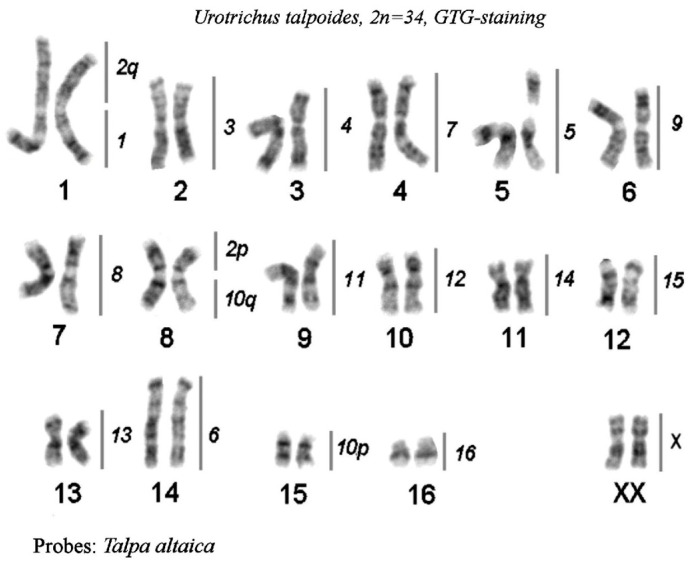
Comparative chromosome map between the Japanese shrew mole, *Urotrichus talpoides*, (2n = 34) and *T. altaica*. The lines and numbers on the right delineate homologous chromosome segments revealed by FISH mapping of the set of *T. altaica* chromosome painting probes on GTG-stained chromosomes of *U. talpoides*. *T. altaica* chromosomes 2 and 10 are split in *U. talpoides*; the *T. altaica* chromosome segments 2p and 10q are fused in chromosome 8 of the *U. talpoides*.

The hybridization results are presented as a map on the G-banded karyotype of the Japanese shrew mole, *Urotrichus talpoides* (Figure 6). Siberian mole painting probes delineated 19 homologous segments in the Japanese shrew mole karyotype. Fifteen Siberian mole chromosomes (TALT 1, 3–9, 11–16, and X) were conserved in toto in the karyotype of *U. talpoides*. Two Siberian mole metacentrics—TALT 2 (Figure 4c) and TALT 10—were divided into two segments, which fused with other elements producing the largest metacentric UTAL 1, medium-size metacentric UTAL 8, and acrocentric UTAL 15.

### 3.4. Comparison of Karyotypes of Four Species from the Talpinae Subfamily

**Figure 7:**
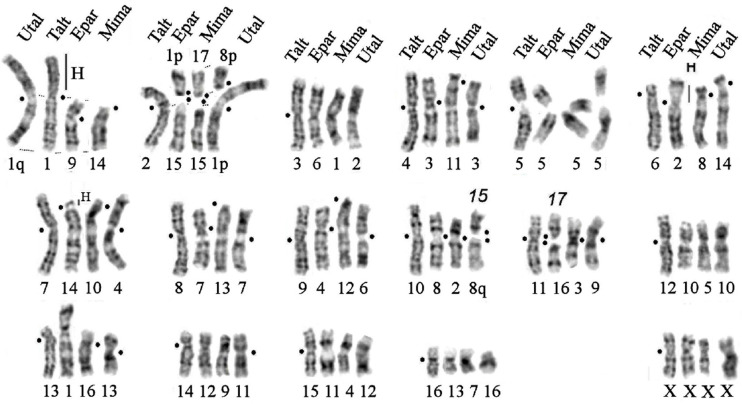
Cross-species chromosome comparison of four talpine species based on chromosome painting of *T. altaica* probes: Japanese shrew mole (*Urotrichus talpoides*, Utal, 2n = 34), Siberian mole (*Talpa altaica*, Talt, 2n = 34), small-toothed mole (*Euroscaptor parvidens*, Epar, 2n = 36), and small Japanese mole (*Mogera imaizumi*, 2n = 36, Mima). “H”—designates constitutive heterochromatin blocks. Black dots designate a centromere position.

The results of painting with flow-sorted chromosome-specific probes of the Siberian mole allowed us to compare species from the other genera of the Talpinae subfamily: mole species from four genera of the Talpini tribe, *Euroscaptor*, *Oreoscaptor*, *Mogera*, and *Talpa* with the Japanese shrew mole (*Urotrichus talpoides*) from the Urotrichini tribe. Figure 7 shows a comparative analysis of the chromosomes of *E. parvidens* (EPAR), *M. imaizumii* (MIMI), and *U. talpoides* (UTAL) ordered relative to the karyotype of the Siberian mole *T. altaica* (TALT).

- Six pairs of autosomes homologous to chromosomes 3, 5, 12, and 14–16 of the Siberian mole remained conserved, as did the X chromosomes in all species;
- Amplification of additional heterochromatin occurred in the p-arm of EPAR 2. A small block of centromeric heterochromatin is present in homologs from all compared species;
- Five pairs of autosomes homologous to chromosomes 4, 7–9, and 13 of the Siberian mole differ by pericentric inversions and/or centromeric shifts;
- Four pairs of autosomes homologous to chromosomes 1, 2, 10, and 11 of the Siberian mole are involved in chromosome fusion/fission.

All obtained data are summarized in Table S2.

### 3.5. Comparison of Karyotypes of Eight Species from the Subfamily Talpinae 

**Figure 8:**
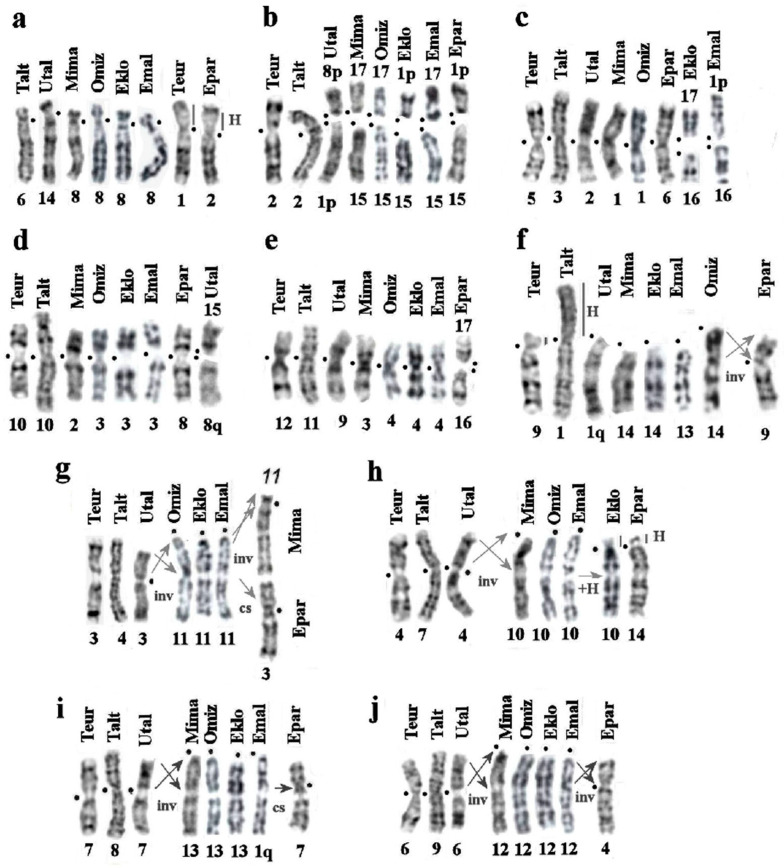
Evolutionary chromosome changes (**a**–**j**) in the complex of eight species of subfamily Talpinae revealed by comparative chromosome painting and G-banding analysis: Siberian mole (*Talpa altaica*, Talt, 2n = 34), European mole (*Talpa europaea*, Teur, 2n = 34), Japanese shrew mole (*Urotrichus talpoides*, Utal, 2n = 34), small Japanese mole (*Mogera imaizumi*, 2n = 36, Mima), Japanese mountain mole (*Oreoscaptor mizura*, Omiz, 2n = 36), Kloss’s mole (*Euroscaptor klossi*, Eklo, 2n = 36), Malaysian mole (*Euroscaptor malayana*, Emal, 2n = 36), and small-toothed mole (*Euroscaptor parvidens*, Epar, 2n = 36). “H”—designates constitutive heterochromatin blocks. Black dot designates a centromere position.

We expanded the number of analyzed species to eight by supplementing the above-mentioned species with the karyotype of the European mole (*Talpa europaea*), previously studied by comparative painting with human probes. Thus, comparing the karyotypes of seven species of the tribe Talpini and one species of the tribe Urotrichini, we tried to find their conserved elements and chromosome rearrangements that accompanied the divergence of these species. Based on the data obtained, we can assume the following (Table 2, Figure 8).

- Five autosomal pairs homologous to chromosomes 5, 12, and 14–16 and the X chromosome of the Siberian mole remained unchanged in all eight mole species;
- Chromosomal pairs homologous to TALT 6 are characterized by the amplification of heterochromatic blocks in the centromeric regions of chromosomes TEUR 1p and EPAR 2p (Figure 8a). Small blocks of heterochromatin are present in the centromeric region of all studied species;
- Whole arm homology is generally preserved:
  (a) UTAL 1p/8p, OMIZ 15/17, MIMI 15/17, EKLO 1p/15, EMAL 15/17, and EPAR 1p/15 to TALT 2/TEUR 2 (Figure 8b);
  (b) EKLO 16/17 and EMAL 1p+16 are homologous to TALT 3 (Figure 8c);
  (c) UTAL 8q/UTAL15 is homologous to TALT 10 (Figure 8d);
  (d) EPAR 16/EPAR 17 is homologous to TALT 11 (Figure 8e).
- Inversions/centromeric shifts occurred on six chromosomes:
  (a) The chromosomal arms TALT 1q, TEUR 9q, and UTAL 1q are homologous to the acrocentrics EKLO 14, EMAL 13, MIMA 14, and OMIZ 14, respectively. Pericentric inversions of the proximal part of the q-arms produced submetacentric chromosomes EPAR 9 and MIMI 14. The short arms of TALT 1 and TEUR 9 are composed of heterochromatic blocks. UTAL1 resulted from a centric fusion of two ancestral acrocentrics (Figure 8f).
  (b) Chromosomes TALT 4, TEUR 3, and UTAL 3 are homologous; pericentric inversions of their p-arms led to the appearance of acrocentric chromosomes 11 in EKLO, OMIZ, and EMAL. A subsequent pericentric inversion of the subcentromeric region led to the appearance of the submetacentric chromosome MIMA 11. A centromeric shift probably led to the appearance of the submetacentric chromosome EPAR 3 (Figure 8g).
  (c) Chromosomes TALT 7, TEUR 4, and UTAL 4 are homologous. Pericentric inversions of the p-arms led to the appearance of acrocentric chromosomes OMIZ 10, EMAL10, and MIMA 10. The appearance of additional heterochromatic arms on EKLO 10 and EPAR 14 explains their subtelocentric morphology (Figure 8h).
  (d) Chromosomes TALT 8, TEUR 7, and UTAL 7 are homologous; pericentric inversions of the p-arm lead to the appearance of acrocentric chromosomes MIMA 13, OMIZ 13, EKLO 13, and q-arm EMAL1. The appearance of the submetacentric EPAR 7 can be explained by the centromeric shift followed by an inversion (Figure 8i).
  (e) Chromosomes TALT 9, TEUR 6, and UTAL 6 are homologous; pericentric inversions of the p-arms led to the appearance of acrocentric chromosomes MIMA 12, OMIZ 12, EKLO12, and EMAL12. A subsequent pericentric inversion of the proximal part of an ancestral acrocentric led to the appearance of a submetacentric EPAR 4 (Figure 8j).
  (f) The most confusing scenario of rearrangements relates to TALT 13, TEUR 13, UTAL 13, MIMA 16, and EPAR 1: the homology of these elements was shown only by the TALT 13 painting probes, and the difference in the GTG pattern of these chromosomes can be explained by a series of inversions. For example, the chromosomes TALT 13 and TEUR 13, according to human painting probe localization and GTG-banding, differ by a pericentric inversion on TEUR 13q and the proximal part of TALT 13q. In *Euroscaptor* species, chromosomes EPAR 1 and EKLO 1 are similar, and both have resulted from fusions of ancestral acrocentrics, whereas their q-arms are homologous to TALT 13 and OMIZ 16. EMAL 1 is also a result of the ancestral centric fusion of two acrocentrics. Thus, TALT 13 homologs in all species have undergone multiple inversions and fusions.

## 4. Discussion

In the latest decades, comparative chromosome painting studies have provided detailed descriptions of tendencies of karyotype evolution in a variety of Mammalian taxa and beyond. However, just a few Eulipotyphla species were covered by chromosome painting, and no detailed painting studies were conducted for a range of Eulipotyphla studies from a particular species group. Here, we remedy this deficiency by studying multiple mole species from two different tribes of the Talpinae family. 

Evolutionary chromosome changes may oftentimes serve as unique cladistic markers, along with craniological and other genetic characteristics, as a basis for species status ranking and as additional support in phylogenetic reconstructions. The use of painting probes expands the possibilities of interspecific comparison and facilitates linking to well-characterized genomes. Although comparative banding studies of talpin moles were conducted previously for multiple species, here we are linking these studies together by constructing chromosome painting maps of key species in two tribes of Talpini. 

### 4.1. Chromosomal Rearrangements in the Genus Euroscaptor

The moles of the Euroscaptor genus inhabit forests and meadows of East and Southeast Asia and it is the second largest genus in Talpinae. Its taxonomy and phylogeny remain controversial. Currently, nine species are included in the genus Euroscaptor, with a separate status of the monospecific genus Oreoscaptor endemic to Japan. The genus Euroscaptor is divided into Western and Eastern clades. Differentially banded karyotypes were available only for three species of this group: two species from the Western clade—*E. klossi* and *E. malayana* (the latter was previously called *E. micrura* malayana)—and the Japanese mountain mole, *Oreoscaptor mizura* [16,23,35], formerly treated as *E. mizura* and recently proposed to be elevated to the genus level [2,4,10,11,36]. By including the endemic Vietnam species *E. parvidens* in the comparative analysis, we not only increased the number of studied karyotypes in the genus but also expanded the representativeness of the analysis by incorporating a member of the Eastern clade. Our findings indicate that each stage of species divergence within the genus is characterized by specific rearrangements in half of the autosomes which are distinct from the conserved autosomes (homologs EPAR 5, 8, 10–15) (Table 1, Figure 3). The karyotype of *O. mizura* differs from that of the Euroscaptor by the absence of evolutionary rearrangements, indicating that its chromosome set is the most conservative. Additionally, the similarity between the karyotypes of *O. mizura* and *M. wogura*, a species from another genus [16], suggests that these species retained a chromosomal set derived from an ancestor common to both genera. The differences between karyotypes of *O. mizura* and *E. malayana* are explained by a reciprocal translocation in the Malaysian mole according to Kawada [35]. Our analysis of a larger number of species revealed a successive occurrence of interchromosomal rearrangements, such as fissions and fusions.

The case of the ancestral chromosome 1 requires particular attention. *O. mizura*, similar to *E. parvidens* and other representatives of the genera *Talpa* and *Urotrichus*, preserved the ancestral variant of chromosome 1 (Table 1 and Table 2, Figure 3 and Figure 8). *E. malayana*, as well as another representative of the Western Euroscaptor clade, *E. klossi*, underwent a fission of the ancestral variant of chromosome 1, which can be considered a karyotypic signature of the Western clade (EKLO 16-17/EMAL 1p-16). Further fusion of one of the formed segments with a large acrocentric in *E. malayana* led to the appearance of the largest submetacentric EMAL 1, which is the distinctive characteristic of the Malaysian mole karyotype. 

The karyotype of the small-toothed mole turned out to be the most rearranged relative to the other species of the genus. The rearrangements include pericentric inversions of four pairs of chromosomes (EPAR 3, 4, 7, and 9), fission that led to the formation of two chromosomes (EPAR 16 and 17), and amplification of heterochromatin on the short arm of EPAR 2. Chromosomal analysis of the remaining species/subspecies of the clade will demonstrate if these rearrangements are characteristic of the entire Eastern clade. According to Zemlemerova et al. [4], there is a large difference in genetic and morphological data between geographically distant populations of *E. parvidens*, in contrast to the closely related species *E. subanura* with low genetic and morphological variability, despite its widespread distribution associated with its recent colonization.

A chromosomal rearrangement, which occurred independently in some species of the Western and Eastern clades (homoplasy), may be a fusion that led to the formation of chromosome 1 in *E. klossi* and *E. parvidens*, but it does not exist in the karyotypes of the Malaysian mole and Japanese mountain mole. Further karyotypic analysis with the involvement of a larger number of species will help to resolve this issue.

The analysis of *Euroscaptor* karyotypes has shown that pericentric inversions, fissions, fusions, and additional heterochromatin play a major role in the evolution of the genus. The chromosome set of the Japanese mountain mole (*O. mizura*) can be considered the closest to the ancestral karyotype of the genus. Significantly, all stages of divergence of the genus carry chromosomal markers for each species and group of species. The inclusion of a larger number of species in the analysis will help to reconstruct the karyotypic history of the genus in more detail.

### 4.2. Chromosomal Rearrangements in the Talpini and Urotrichini Tribes

We expanded the comparative analysis of karyotypes to the level of a subfamily, by including eight species representing not only the genera *Euroscaptor* and *Oreoscaptor* but also the genera *Mogera* and *Talpa* from the Talpini tribe and used *Urotrichus talpoides* from the Urotrichini tribe as an outgroup. For comparison, the chromosomal painting was performed with sorted chromosomes of the Siberian mole (*T. altaica*) on chromosomes of *E. parvidens, M.ogera imaizumii*, and *U. talpoides*. The comparison was supplemented by the above-considered species of the genus *Euroscaptor* and the European mole *T. europaea* described earlier [26,27].

The analysis showed that autosomes, homologous to the large NOR-bearing chromosome (TALT 5) and most of the small autosomes (TALT 12, 14–16), as well as the X chromosome, remained unchanged in all eight mole species (Table 2, Figure 8). Variations in the amount of heterochromatin on TALT 1 and TEUR 9 homologs are found only in species of *Talpa*, including *T. romana* and *T. occidentalis* [21,37], as is the TALT 2p+2q/TEUR 2p+2q fusion. Both of these characteristics can serve as cytogenetic signatures of the genus *Talpa*.

The rate of chromosomal rearrangements is significantly higher in the Asian mole genera. Chromosomal analysis revealed that the pericentric inversions of four pairs of chromosomes (homologous to the chromosomes of the Siberian mole 4, 7, 8, and 9) are common to two closely related genera, *Mogera* and *Euroscaptor* (Table 2, Figure 7 and Figure 8). The presence of common chromosomal characteristics is consistent with morphological and genetic data on their close relationship. 

Interestingly, further rearrangements of these chromosomes are observed in representatives of both genera. Thus, the homologs of the TALT 4 chromosome subsequently underwent an inversion in *M. imaizimii* (chromosome 11) and a centromeric shift in *E. parvidens* (chromosome 3). It should be noted that it is this inversion that separates the karyotypes of *M. imaizimii* and *M. etigo* from the karyotype of *M. wogura* [16], and perhaps it is an apomorphic trait of the *M. imaizimii* karyotype, while the other species of *Mogera* preserved a common variant of chromosome 11 for both genera. TALT 7 homologs in *E. parvidens* (chromosome 14) and in *E. klossi* (chromosome 10) developed small heterochromatic short arms. According to Kawada [16], four inversions on homologs of TALT 7, TALT 8, TALT 13, and TALT 2p separate the Korean *M. robusta* and Japanese populations of *M. wogura*. TALT 8 homologs in *E. parvidens* (chromosome 7) and *E. malayana* (chromosome 1) underwent a centromeric shift and a fusion, respectively. The TALT 9 homolog has undergone another inversion in *E. parvidens* (chromosome 7). In general, for each species in the genus *Euroscaptor*, there is one characteristic feature associated with further chromosome transformations homologous to one of these four chromosomes. The exception is *E. parvidens*, in which all four homologous chromosomes underwent further rearrangements and additional fission, which led to the formation of chromosomes EPAR 16 and EPAR17. At the moment, the karyotype of the small-toothed mole is the most derived among all the mole species studied so far. 

It can be assumed that the karyotype of the Western form of *U. talpoides*, used here, is the closest to the ancestral karyotype of the Urotrichini tribe. According to our data, the phylogenetic features of the genus *Urotrichus* include two fusions that led to the formation of UTAL 1 and UTAL 8 and one fission that led to the formation of UTAL 15 and UTAL 8q (Table 2, Figure 8). It is noteworthy that the accumulation of heterochromatin is characteristic of UTAL 14 homologs, which is not shown for the Western form but is observed together with a pericentric inversion in the Eastern form of the species.

The evolution of Talpini karyotypes was accompanied by fusion–fission events, inversions, centromeric shifts, and heterochromatin expansion. Urotrichini karyotypes were formed through the fusions and fission of ancestral chromosomes. 

### 4.3. Reconstruction of the Ancestral Karyotype of the Talpini and Urotrichini Tribes

Our analysis allowed us to propose a possible ancestral karyotype of the tribe and, based on it, to trace the features of chromosomal rearrangements accompanying the divergence of moles. The existing phylogenetic trees of moles differ in the position of the Japanese mountain mole *Oreoscaptor mizura* relative to representatives of the genera *Mogera* and *Euroscaptor*. Some researchers place it basal to these genera [3,4,10,11] and others place it in a separate clade together with *Mogera*, different from the branch leading to *Euroscaptor*, *Parascaptor,* and *Scaptochirus* [2,7].

**Figure 9:**
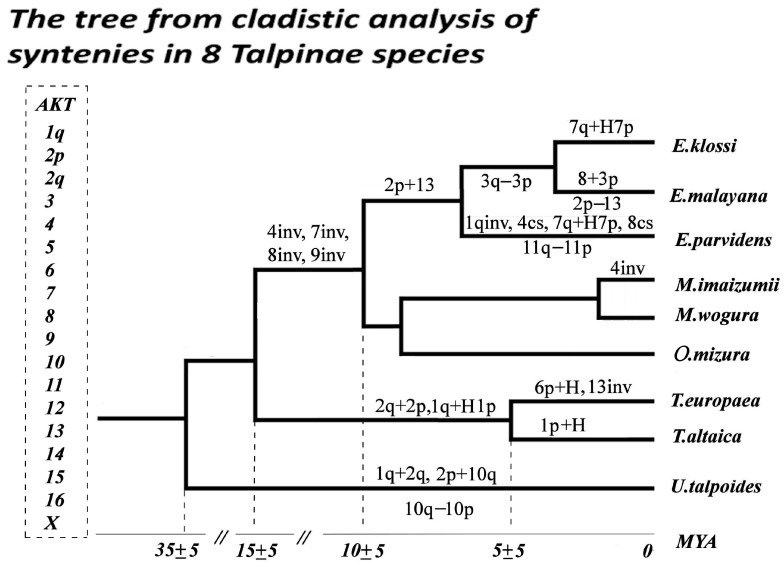
The phylogenetic tree based on cladistic analysis of ancestral chromosome syntenies in a complex of eight Talpinae species from genera *Euroscaptor*, *Oreoscaptor*, *Mogera*, *Talpa*, and *Urotrichus*. inv—inversion, cs—centromeric shift, ‘+’—fusion, ‘-’—fission, “H”—additional heterochromatic block, AKT—Ancestral Karyotype of Talpinae. MYA—million years ago. Divergence dates are from He et al. [2].

During the analysis of the numbers of chromosomal rearrangements on the phylogenetic tree, we observed different rates of karyotypic transformations (Figure 9). Two rearrangements, including one fusion and amplification of heterochromatin, took place in about 10 million years of the shared history of the Siberian and European moles. Over the next 5 million years after this divergence, *T. europaea* underwent a pericentric inversion in chromosome 13 and an amplification of heterochromatin in the short chromosome 1, and *T. altaica* only acquired an amplified heterochromatic block in the short arm of chromosome 1. The data obtained confirm chromosomal conservation within the genus *Talpa*, which also demonstrates morphological stability as opposed to high genetic variability [7]. According to our results, only the karyotype of *Urotrichus talpoides* from the closely related Urotrichini tribe is more stable, with three rearrangements over 35 million years. 

Karyotypes of the Asian species were actively rearranged after divergence from the genus *Talpa*, undergoing four inversions over 5 million years; then, the rate of karyotypic divergence became heterogeneous in different lineages. Exceptional chromosome conservation is noted in the Japanese mountain mole *O. mizura*, which is consistent with its basal position relative to the rest of the Asian moles (*Euroscaptor, Mogera*). The karyotype of the Japanese *M. wogura* is no less stable, whereas the Korean population of *M. robusta* underwent four inversions in a fairly short time after divergence [16]. Karyotypes of representatives of the Western clade of *Euroscaptor (E. klossi and E. malayana*) underwent only a few rearrangements, while further divergence of the Eastern clade (*E. parvidens*) was accompanied by five rearrangements over about 7 million years.

The data obtained here by karyotype analysis of the species from the Talpini and Urotrichini tribes allowed us to estimate the rates of chromosomal transformations within the Talpinae subfamily. A high level of chromosomal conservation in the genus *Talpa* was confirmed, and cytogenetic signatures in each of the species groups were determined. It is shown that the Asian species of the tribe are characterized by pericentric inversions (and other transformations) of four pairs of autosomes. The karyotype of the Japanese mountain mole *O. mizura* seems to be the most conserved among the Asian moles. The most frequently occurring types of chromosomal rearrangements in moles are the pericentric inversions and the amplification of heterochromatin. The inclusion of a larger number of species in the comparative analysis will allow us to reconstruct the chromosomal history of moles more accurately.

The set of chromosome-specific pools obtained here can be further sequenced [38] and used for mole C-scaffold assemblies in large-scale genome sequencing projects [39].

## Figures and Tables

**Table 1:** Correspondence between chromosomes of the *Euroscaptor* species arranged according to the *Oreoscaptor mizura* chromosomes. H—constitutive heterochromatin, inv—inversion.

*O. mizura*	*E. parvidens*	*E. klossi*	*E. malayana*
1	6	16+17	1p+16
2	5	2	2
3	8	3	3
4	16+17	4	4
5	10	5	5
6 q H	11+q H	6+H	6+H
7	13	7	7
8	2+p H	8	8
9	12	9	9
10 q H	14+p H	10+p H	10+q H
11	3 inv	11	11
12	4 inv	12	12
13	7 cs	13	1q
14	9inv	14	13
15	15	15	15
16	1q	1q	14
17	1p	1p	17

**Table 2:** The correspondence between chromosomes of Talpinae species. inv—inversion, cs—centromeric shift, H—constitutive heterochromatin.

*T. altaica*	*T. europaea*	*U. talpoides*	*O. mizura*	*M. imaizumii*	*E. klossi*	*E. malayana*	*E. parvidens*
1q (+p H)	9q (+p H)	1q	14	14	14	13	9 inv
2	2	1p+8p	17+15	15+17	1p+15	17+15	1p+15
3	5	2	1	1	16+17	1p+16	6
4	3	3	11 inv	11 inv+inv	11 inv	11 inv	3 inv+cs
5	8	5	2	5	2	2	5
6q	1+p H	14	8q	8q	8q	8q	2+p H
7	4	4	10 inv	10 inv	10 inv+p H	10 inv	14 inv+p H
8	7	7	13 inv	13 inv	13 inv	1q inv (+1p)	7 inv+cs
9	6	6	12 inv	12 inv	12 inv	12 inv	4 inv+inv
10	10	8q+15	3	2	3	3	8
11	12	9	4	3	4	4	16+17
12	11	10	5	5	5	5	10
13	13 inv	13	16	16	1q	14	1q
14	14	11	9	9	9	9	12
15	15	12	6	4	6	6	11
16	16	16	7	7	7	7	13

*T. altaica*

*T. europaea*

*U. talpoides*

*O. mizura*

*M. imaizumii*

*E. klossi*

*E. malayana*

*E. parvidens*

## Data Availability

All data in the article.

## Supplementary Materials

The following supporting information can be downloaded at: https://www.mdpi.com/article/10.3390/genes14071472/s1.
