# Supplementary material for: Chromosomal Evolution of the Talpinae
Source: Genes (Basel). 2023 Jul 19;14(7):1472. doi: 10.3390/genes14071472 (PMC10379030; doi:10.3390/genes14071472)
Supplement: Supplementary file 1 [file genes-14-01472-s001.zip › Table S2.pdf]

Supplement. Table S2. The chromosomes of the Talpinae species arranged according to the *Talpa altaica* chromosomes analyzed by chromosome painting

Talpa altaica	***Euroscaptor parvidens***	***Mogera imaizumii***	***Urotrichus talpoides***
1	9	14	1q
2	1p+15	15+17	1p+8p
3	6	1	2
4	3	11	3
5	5	5	5
6	2	8	14
7	14	10	4
8	7	13	7
9	4	12	6
10	8	2	8q+15
11	16+17	3	9
12	10	5	10
13	1q	16	13
14	12	9	11
15	11	4	12
16	13	7	16
