# Supplementary material for: Chromosomal Evolution of the Talpinae
Source: Genes (Basel). 2023 Jul 19;14(7):1472. doi: 10.3390/genes14071472 (PMC10379030; doi:10.3390/genes14071472)
Supplement: Supplementary file 1 [file genes-14-01472-s001.zip › Table S3.pdf]

Supplement. Table S3. Corresponding between sorting samples and chromosomes of the *Talpa altaica*

N	*Peak*	*Probe*	*Chromosome*
1	A	1, 2, 3	1
2	I	15, 22	2
3	C	3	3
4	K	9, 18	4+7
5	D	4	5
6	J	8, 13	6+9
7	L	10	8
8	E	5	10
9	F	6	11
10	G	21	12
11	H	7	13+X
12	N	12, 20	14
13	M	11, 19	15
14	B	2	16
