# Supplementary figures and images for: Chromosomal Evolution of the Talpinae
Source: Genes (Basel). 2023 Jul 19;14(7):1472. doi: 10.3390/genes14071472 (PMC10379030; doi:10.3390/genes14071472)

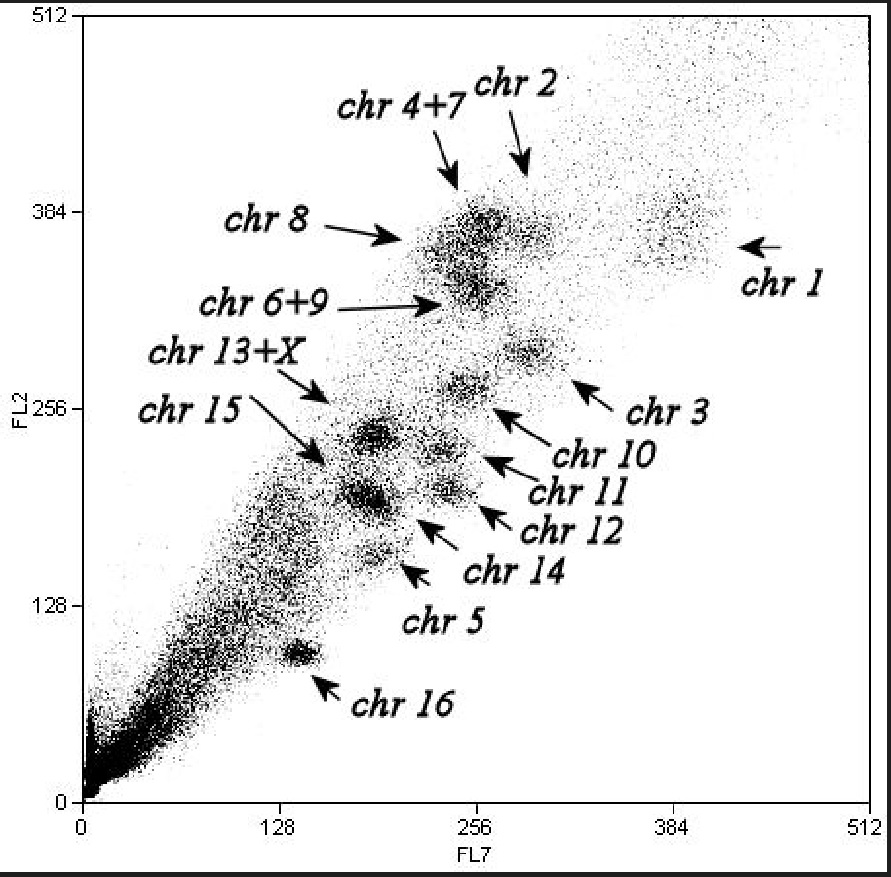

Supplement: Supplementary file 1 [file genes-14-01472-s001.zip › Figure S1new.jpg]

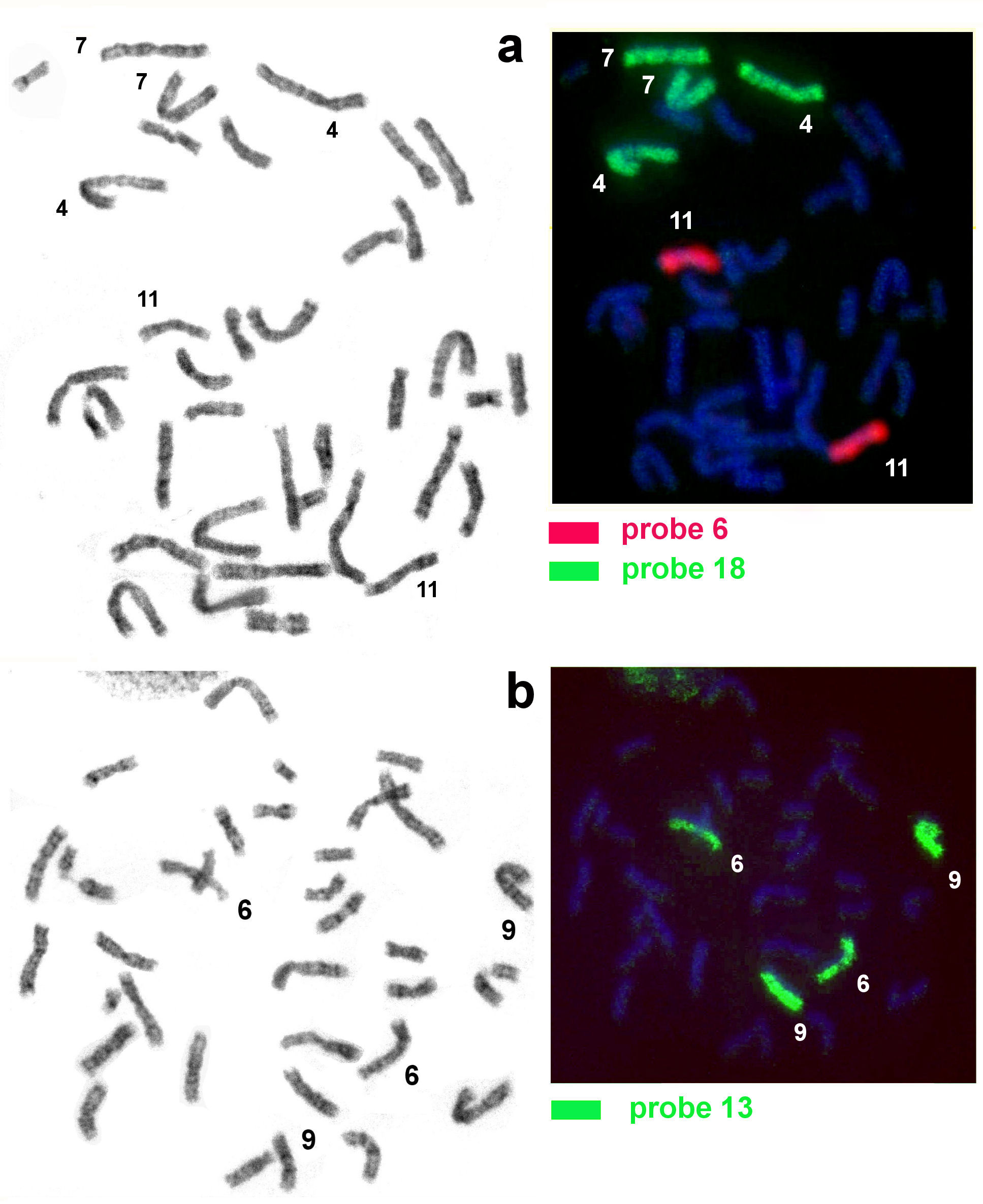

Supplement: Supplementary file 1 [file genes-14-01472-s001.zip › Figure S2.jpg]

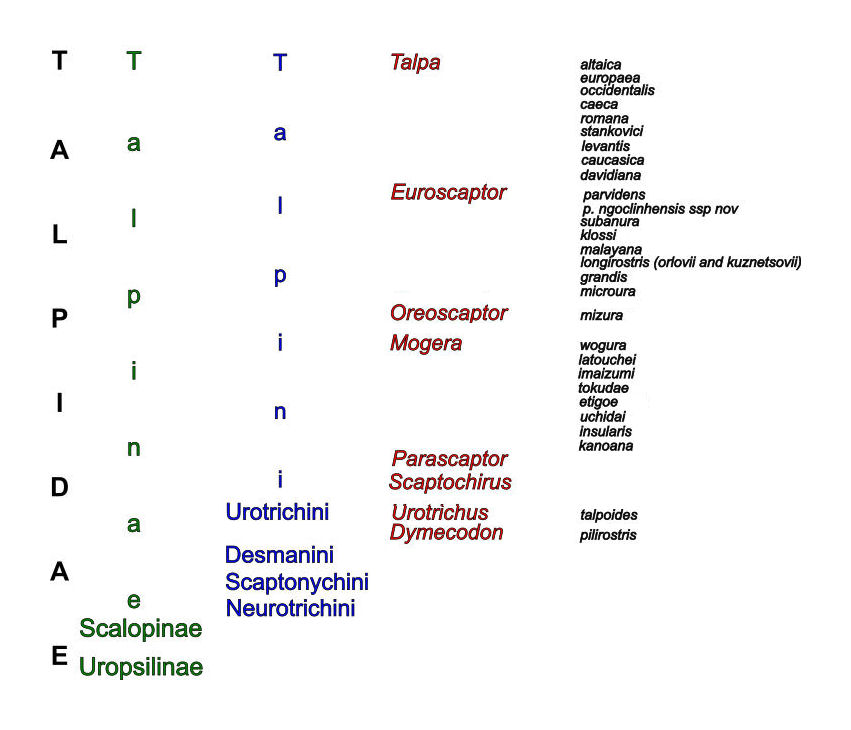

Supplement: Supplementary file 1 [file genes-14-01472-s001.zip › Table S1.jpg]
